# Case Report: A 55 kg retroperitoneal liposarcoma

**DOI:** 10.3389/fonc.2025.1621829

**Published:** 2025-06-18

**Authors:** Ren Yingzheng, An Junjie, Wang Guifei, Yang Yang, Dong Long, Jiang Linlin, Fang Zhiqiang, Bi Xiaogang, Dong Yonghong

**Affiliations:** ^1^ Department of Gastrointestinal, Pancreatic, Hernia and Abdominal Wall Surgery, Shanxi Provincial People’s Hospital, Taiyuan, Shanxi, China; ^2^ Department of Anesthesiology, Shanxi Provincial People’s Hospital, Taiyuan, Shanxi, China; ^3^ Department of Critical Care Medicine, Shanxi Provincial People's Hospital, Taiyuan, Shanxi, China

**Keywords:** retroperitoneal liposarcoma, multidisciplinary team, complication, surgery, prevention

## Abstract

**Background:**

Sarcomas, which are mesenchymal malignancies, account for less than 1% of all cancers. Retroperitoneal liposarcoma (RPLS), particularly the well-differentiated subtype, often presents as large masses due to its deep anatomical location and indolent growth pattern. Moreover, its frequent adherence to vital structures poses significant challenges for complete surgical resection.

**Case presentation:**

A 62-year-old male was admitted to the hospital due to the gradual enlargement of a mass in the abdomen and pelvis over the past 20 years. In the recent five months, the mass has significantly increased in size, leading to compression symptoms such as dyspnea and lower limb edema. Physical examination showed that the abdomen was distended, and varicose veins on the abdominal wall were visible. The peak value of the abdominal circumference reached 165 cm. Abdominal and pelvic CT examination indicated that there was a huge mass with mixed density in the abdominal and pelvic cavity, which contained lipid, calcification, and soft tissue density shadows. The lesion was so huge that it exceeded the scanning field, and its size could not be measured. Subsequently, the patient underwent a resection of the huge retroperitoneal tumor and a combined resection of multiple organs under general anesthesia. The huge retroperitoneal mass, approximately 70*54*20 cm in size and weighing 55 kg, was removed. The postoperative pathology confirmed it as well-differentiated liposarcoma. The surgical process was relatively smooth. However, unfortunately, 40 days after the operation, the patient died of multiple organ dysfunction due to pneumonia, heart failure, and intra-abdominal infection after anastomotic leakage.

**Conclusions:**

While Complete surgical resection (R0) remains the gold standard for RPLS management, radical multivisceral resection of massive tumors requires meticulous evaluation by a multidisciplinary team (MDT), encompassing patient fitness, tumor biology, and perioperative risk stratification. When achieving R0 resection is deemed unfeasible or carries prohibitive risks, staged debulking surgery may be considered as an alternative approach. The application of hyperthermic intraperitoneal chemotherapy (HIPEC) should be evaluated judiciously on a case-by-case basis.

## 1 Introduction

RPLS is a malignant neoplasm originating from adipose tissue in the retroperitoneal space, accounting for approximately 10%-20% of all soft tissue sarcomas (1). This tumor predominantly affects individuals aged 50–70 years, with no significant gender predilection and an estimated annual incidence of 2.5 cases per million population (2). Histologically, RPLS is classified into five main subtypes: well-differentiated liposarcoma (40-50% of cases), dedifferentiated liposarcoma, myxoid liposarcoma, pleomorphic liposarcoma, and pleomorphic myxoid liposarcoma (3). Current diagnostic algorithms primarily incorporate computed tomography (CT) and magnetic resonance imaging (MRI), while histopathological examination remains the gold standard. Molecular analysis of MDM2 and CDK4 gene amplification provides crucial diagnostic confirmation (1). R0 resection with microscopically negative margins represents the cornerstone of treatment, typically requiring en bloc removal of the tumor and involved tissues to minimize recurrence risk (4). Adjuvant therapies such as HIPEC may benefit high-risk patients, particularly those with peritoneal dissemination potential. While retrospective studies demonstrate HIPEC’s efficacy in reducing intraperitoneal tumor implantation and local recurrence rates (5), its application requires careful consideration of individual patient factors, surgical outcomes, and recurrence risk stratification. Tumors exceeding 20 kg are classified as giant liposarcomas, constituting exceptionally rare clinical presentations. We report a notable case of a 55 kg giant RPLS, whose successful resection and postoperative management posed extraordinary surgical challenges, offering valuable insights for similar cases.

## 2 Case presentation

**Figure 1:**
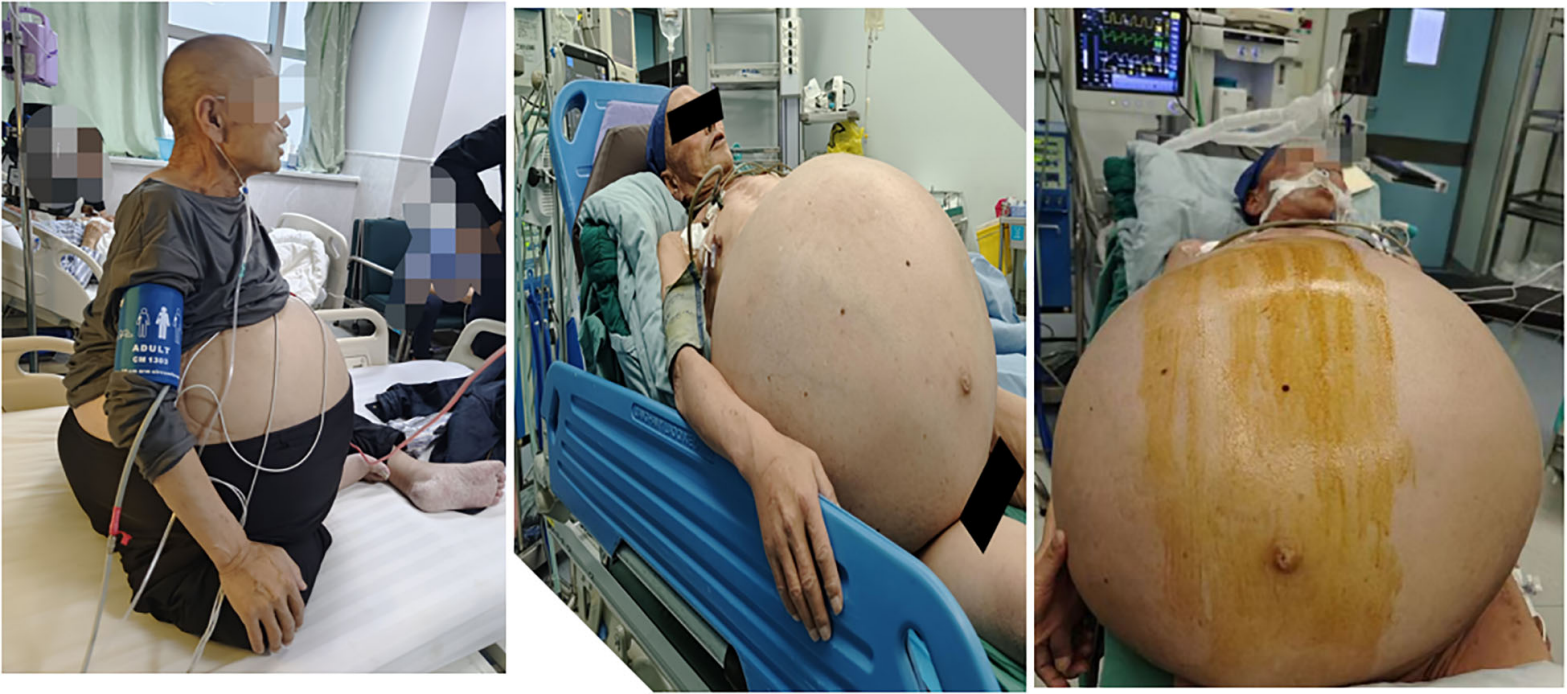
General condition of the patient.

This case report documents a 62-year-old male patient with a complex retroperitoneal tumor manifesting characteristic clinical features. The patient’s medical history revealed progressive abdominal enlargement over two decades, with notable symptom exacerbation in the final five months prior to presentation, and there was no family history of similar tumors or relevant genetic syndromes. This rapid progression was marked by the development of compressive symptoms including resting lower chest tightness, dyspnea, and bilateral lower extremity edema. Physical examination findings were significant for open-mouth breathing pattern and profound abdominal distension measuring 165 cm in circumference (**Figure 1**). Abdominal palpation demonstrated medium firmness without visible intestinal peristalsis, though prominent abdominal wall varicosities were observed. Auscultation revealed hypoactive bowel sounds at 3 per minute.

**Figure 2:**
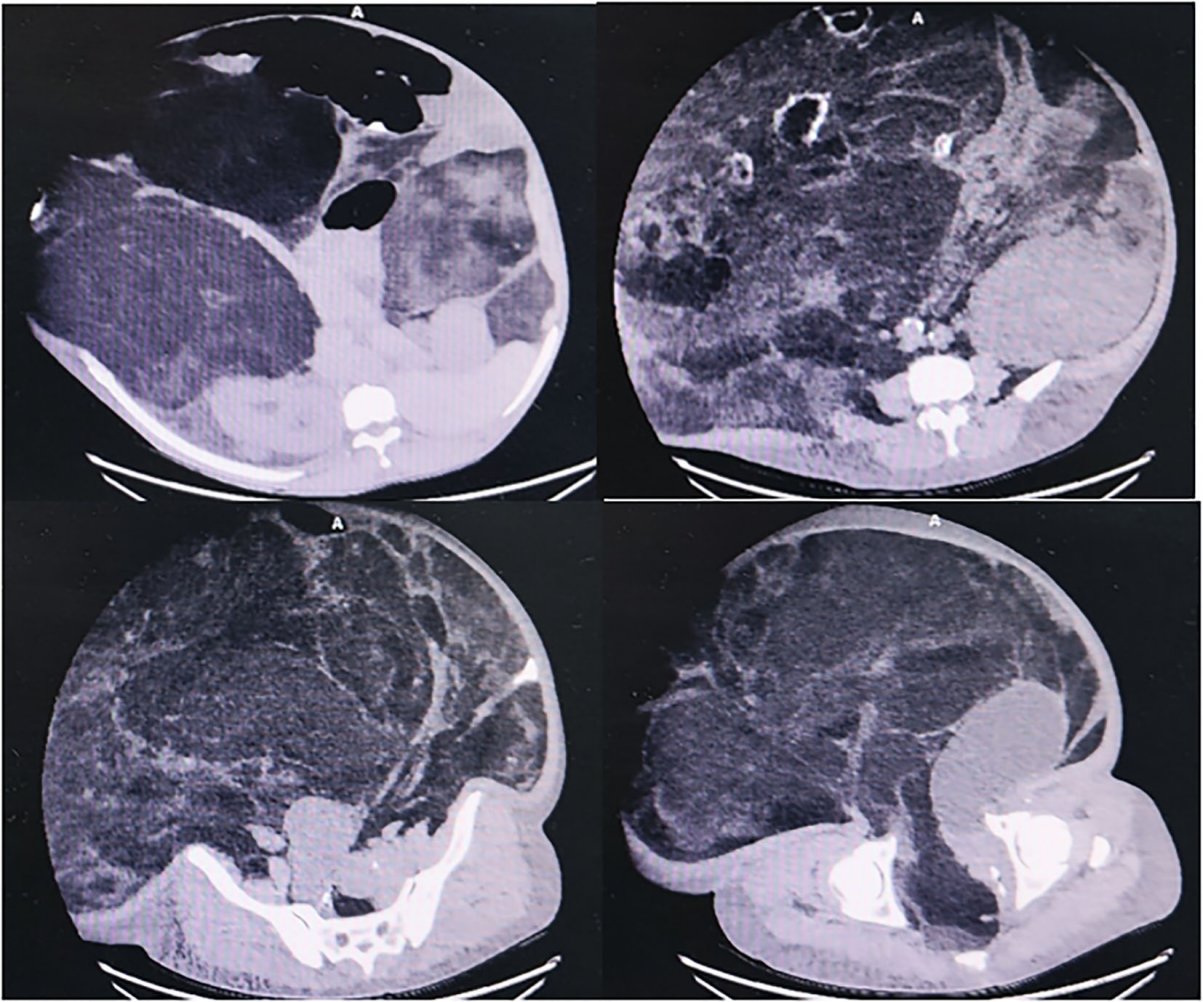
The patient’s CT images.

**Figure 3:**
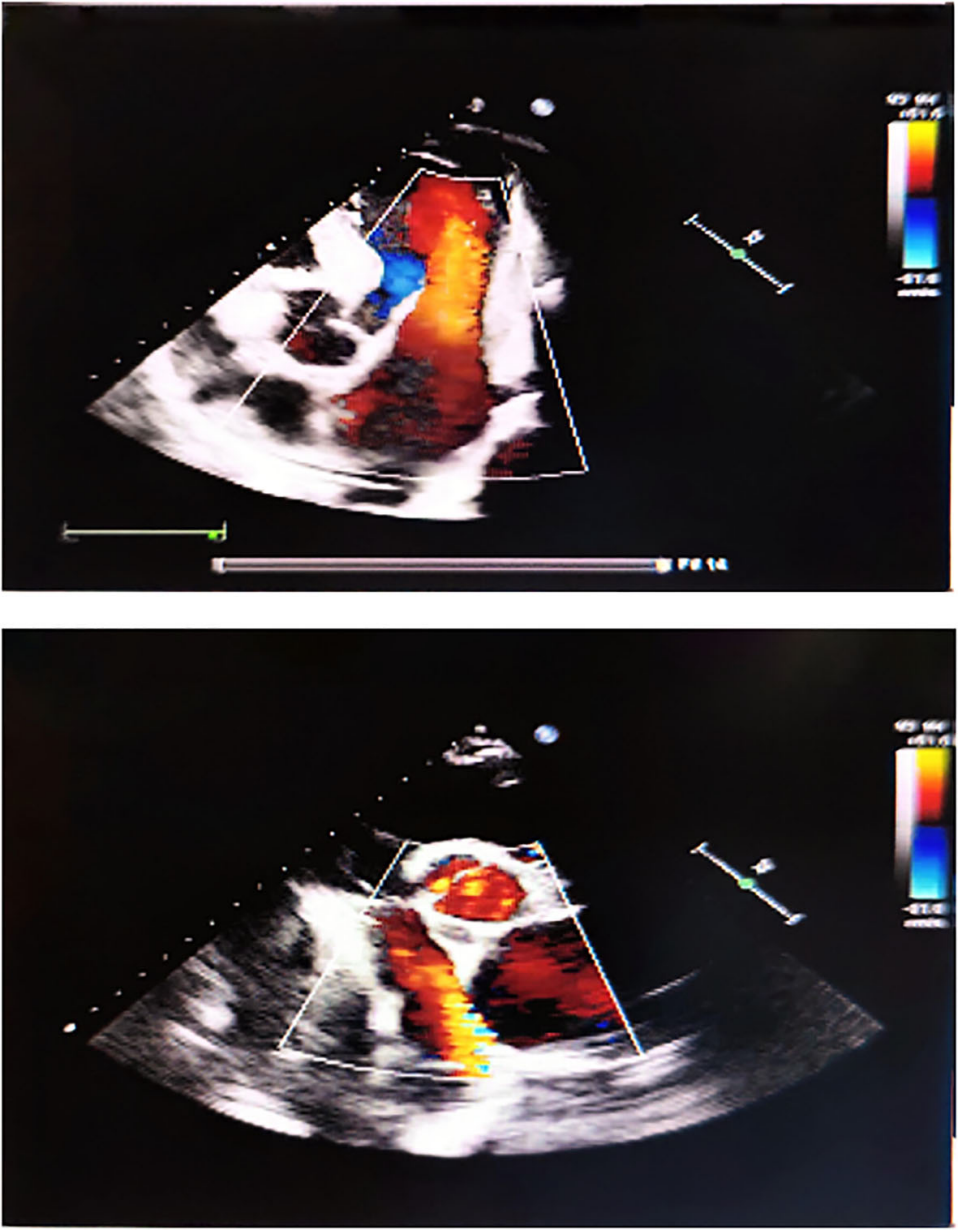
Echocardiography suggests left atrial enlargement.

**Figure 4:**
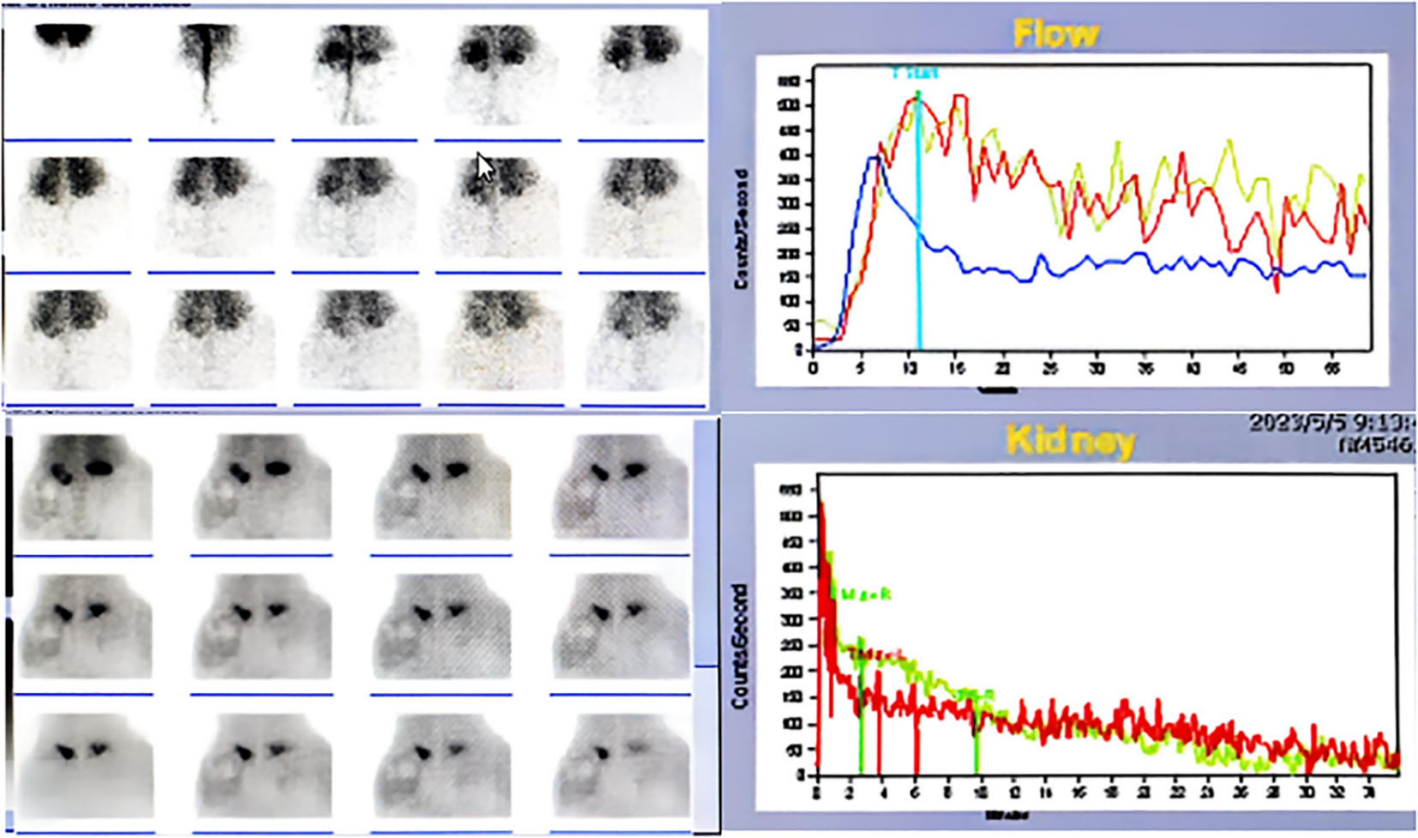
Renal dynamic imaging.

Diagnostic imaging provided critical insights into the tumor’s characteristics and anatomical relationships. Abdominopelvic CT imaging (**Figure 2**) delineated a massive heterogeneous mass containing adipocytic, calcific, and soft tissue components that exceeded the scan field dimensions. The tumor demonstrated intimate proximity to major vascular structures including the abdominal aorta and inferior vena cava. Complementary imaging studies yielded additional findings: echocardiography (**Figure 3**) demonstrated cardiomegaly with left atrial enlargement (46×51×63 mm) and preserved cardiac function (EF 70%, FS 40%, EDV 131ml, ESV 39ml, SV 92ml) with normal valvular morphology and motion, along with bilateral diaphragmatic elevation; chest radiography showed pulmonary vascular congestion; renal dynamic imaging confirmed severe left renal impairment (GFR 24.3 ml/min) with upper urinary tract obstruction evidenced by contrast retention at 20 minutes without excretion after furosemide administration, while the total GFR was maintained at 62.9 ml/min (**Figure 4**).

The patient’s preoperative lab work demonstrated mild respiratory alkalosis (pH 7.515, PaCO_2_29.5 mmHg) with well-maintained oxygenation (PaO_2_98.8 mmHg, SpO_2_98%) and normal lactate (1.94 mmol/L). Laboratory findings revealed mild anemia (Hb 95 g/L) with elevated inflammatory markers (CRP 75 mg/L) but normal WBC (6×10^9^/L) and platelet counts (291×10^9^/L). Coagulation studies were essentially normal except for slightly increased D-dimer (370 ng/mL). Liver and renal function tests, including ALT (13.38 IU/L), AST (20.67 IU/L), albumin (35 g/L), and creatinine (72.5μmol/L), were all within normal limits, as was HbA1c (5.2%). Overall, these results suggested mild anemia and systemic inflammation without evidence of significant organ dysfunction.

**Figure 5:**
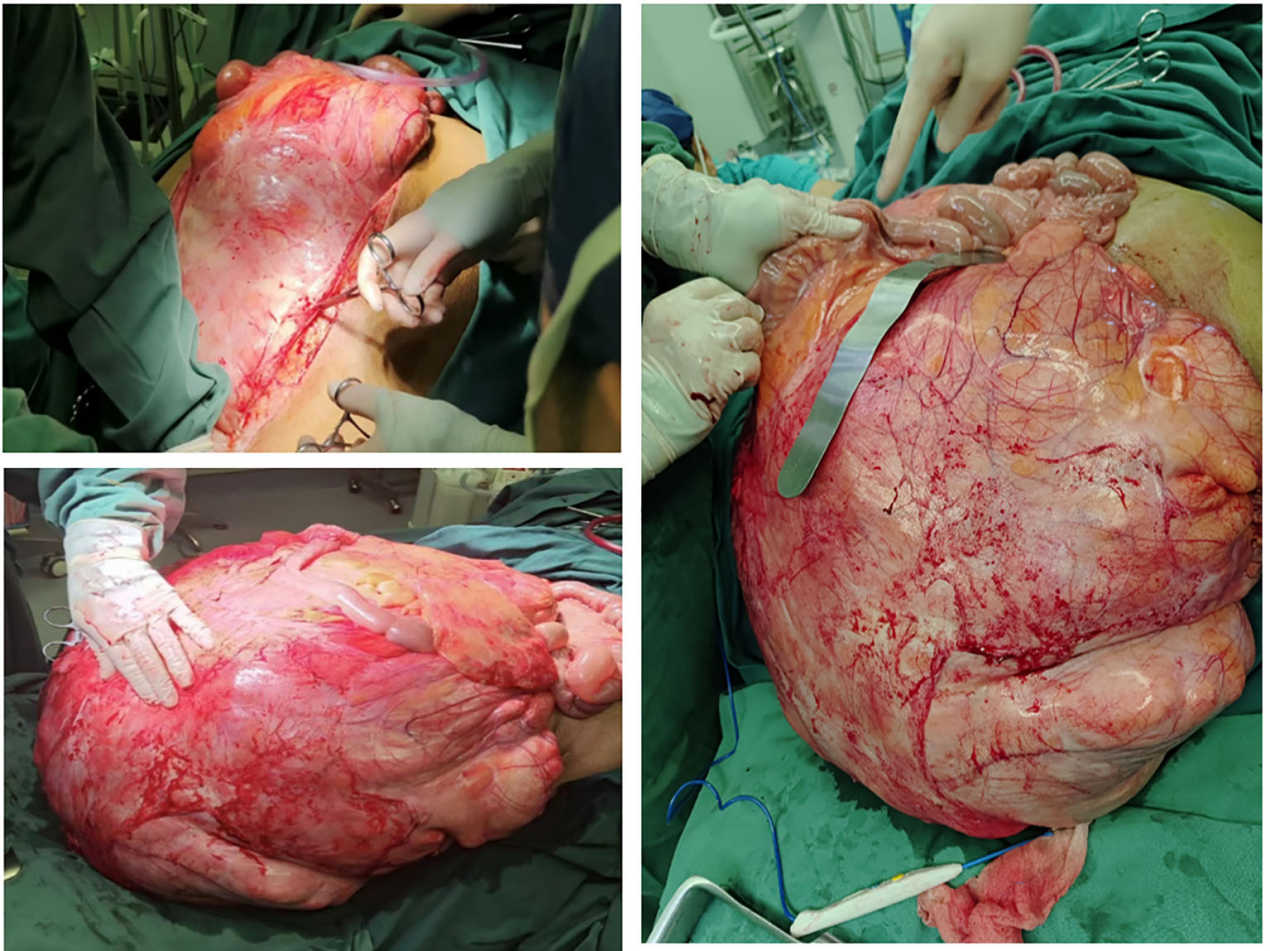
Surgical operation pictures.

**Figure 6:**
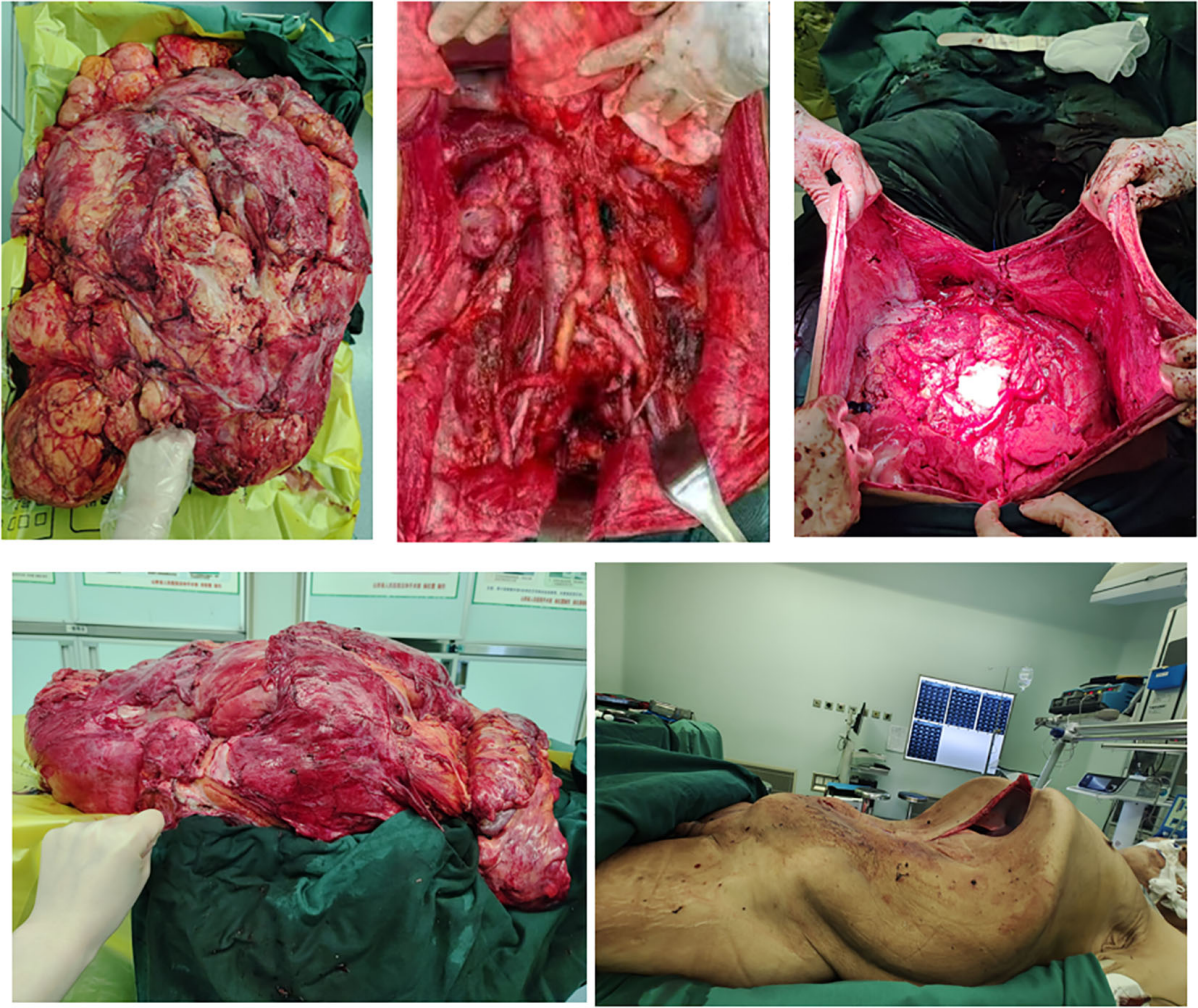
Surgical operation pictures.

The patient presented with severe dyspnea that prevented lying flat. Preoperative pulmonary function assessment showed an mMRC dyspnea grade of 3, meaning the patient needs to stop and catch breath after walking 100 meters or several minutes on level ground. The COPD Assessment Test (CAT) yielded a total score of 25, indicating severe pulmonary dysfunction. These results clearly demonstrate that the patient’s lung function is severely impaired, significantly affecting daily activities and rest. Following comprehensive laboratory and imaging evaluations, a MDT consisting of specialists from gastrointestinal surgery, urology, pulmonology, cardiology, anesthesiology, critical care medicine, radiology, and pathology conducted a joint consultation. After thorough discussions with the patient and family members regarding the anticipated surgical outcomes and potential complications (including but not limited to bleeding, infection, anastomotic leakage, organ dysfunction, and even mortality risks), and upon obtaining fully informed consent, the team ultimately decided to perform “resection of a giant retroperitoneal tumor combined with multivisceral resection” (**Figures 5**, **6**). Preoperative assessment indicated that due to the tumor’s enormous size, tight vascular adhesions, and complex anatomical relationships, needle biopsy would not only fail to obtain representative tissue samples but also carry a high risk of hemorrhage.

**Figure 7:**
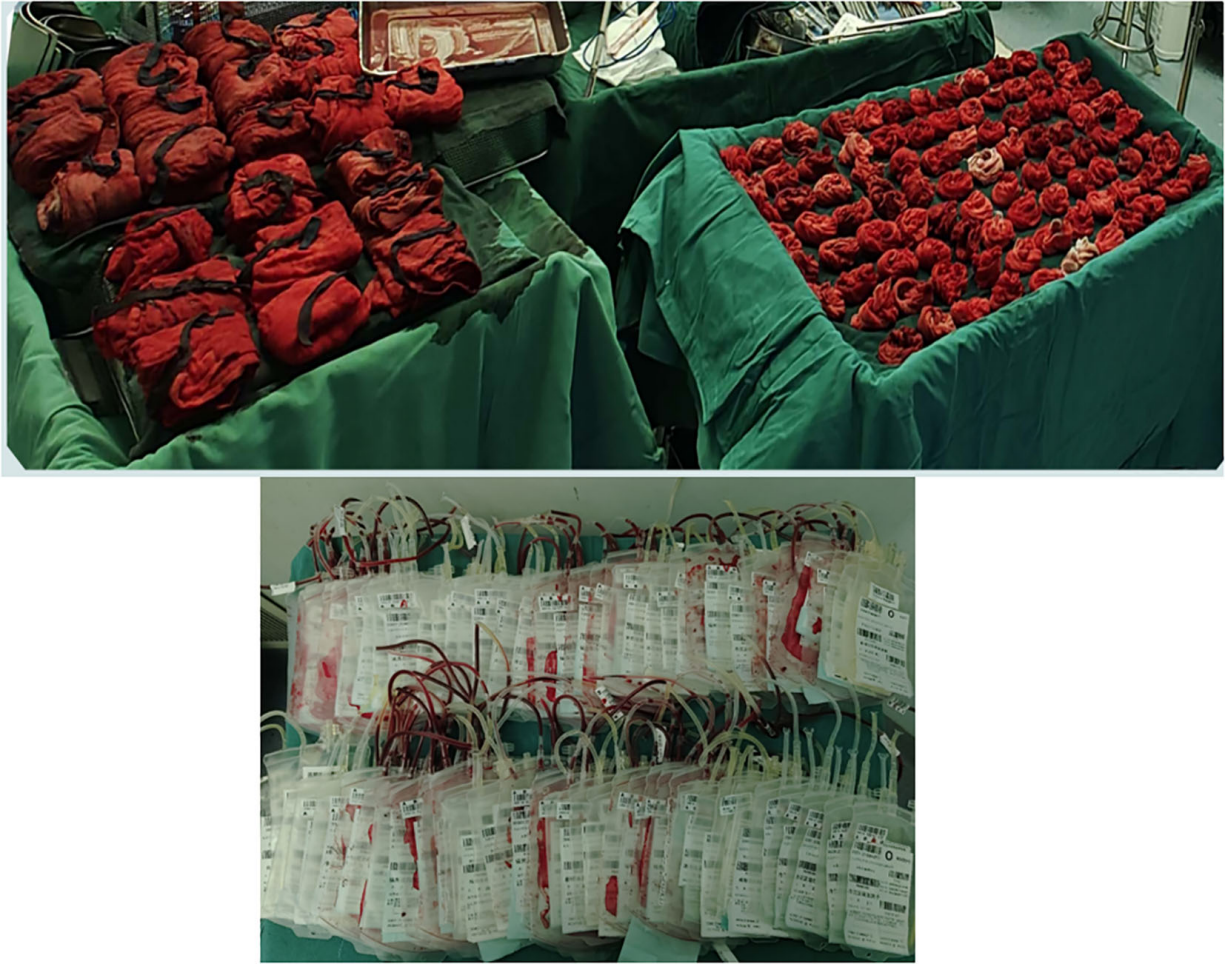
Intraoperative Blood Transfusion Management.

The patient received preoxygenation followed by anesthesia induction. After achieving adequate anesthetic effect, endotracheal intubation was promptly performed. Subsequently, the oxygen saturation gradually dropped to 85% with a sharp rise in airway pressure, resulting in complete mechanical ventilation failure. Following consultation with the anesthesiologist, mechanical ventilation was switched to manual bag ventilation, leading to improvement of oxygen saturation to 94%. The situation was attributed to diaphragmatic compression caused by the massive tumor mass after anesthesia induction, which elevated airway pressure. An emergency laparotomy was therefore immediately performed to relieve thoracic pressure. Intraoperative findings revealed a colossal tumor measuring 70–80 cm in maximal diameter with extensive invasion of multiple abdominal viscera. The mass exhibited particularly tenacious adhesions to critical vascular structures, most notably the inferior vena cava and abdominal aorta. The surgical procedure spanned 26 hours and was strategically divided into three distinct phases. The initial phase focused on upper abdominal tumor resection with meticulous preservation of renal vasculature. Subsequent phases addressed mid and lower abdominal components through tumor isolation, inferior mesenteric artery ligation, and partial resection of bladder and rectal tissues. The final phase encompassed complete lymph node dissection and definitive hemostasis. Concurrent procedures included HIPEC (maintained at 43°C for 90 minutes), ileotransverse anastomosis, diverting transverse colostomy, gastrostomy tube placement, right cutaneous ureterostomy, and bilateral ureteral stent insertion. The patient received prophylactic imipenem during the procedure to prevent infection. Intraoperative vital signs remained stable throughout the operation. Despite substantial blood loss requiring a 10,000 mL transfusion (**Figure 7**), the patient was transferred back to the ward in stable condition postoperatively with maintained hemodynamic stability.

**Figure 8:**
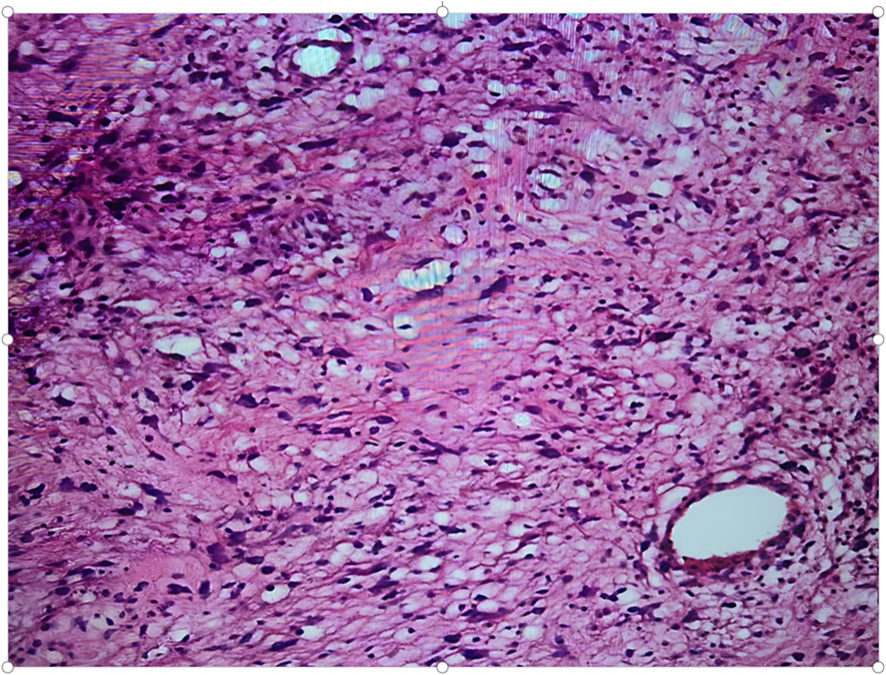
Diagram of the pathological section.

**Figure 9:**
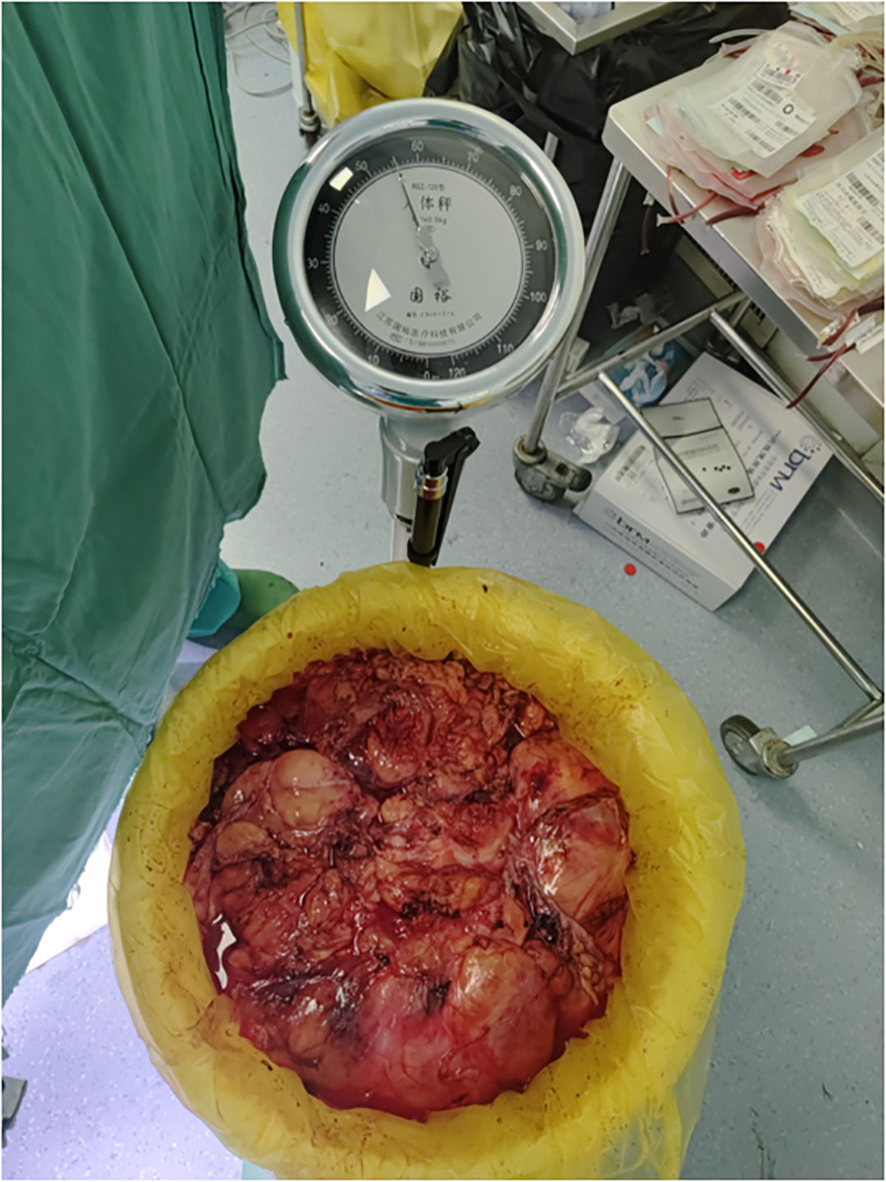
The weight of retroperitoneal liposarcoma.

Pathological examination of the resected specimen (**Figures 8**) documented a 55 kg (**Figures 9**) retroperitoneal tumor measuring 70×54×20 cm. Macroscopic evaluation showed predominantly light yellow cut surfaces with interspersed grayish-white and translucent areas displaying variegated consistency. Multiple satellite nodules were identified, the largest measuring 35×25×14 cm. Histopathological analysis confirmed well-differentiated liposarcoma with focal dedifferentiation, characterized by atypical spindle cells, hyperchromatic nuclei, and necrotic foci. Immunohistochemical profiling demonstrated scattered MDM2 positivity, diffuse CDK4 expression, and elevated Ki-67 proliferative index (20% in hotspots), with molecular confirmation of MDM2 gene amplification. The tumor exhibited extensive local invasion involving rectal serosa (affecting 6.5 cm of intestinal length with 2.5 cm transmural penetration), splenic adhesions (3×2.5×1 cm), and periappendiceal tissues (with coincident acute exacerbation of chronic appendicitis). Incidental findings included a left renal cyst (3.8×2.5×0.1 cm) and a benign fibro-osseous nodule in the left common iliac vein (1.5×1.2×0.4 cm).

**Figure 10:**
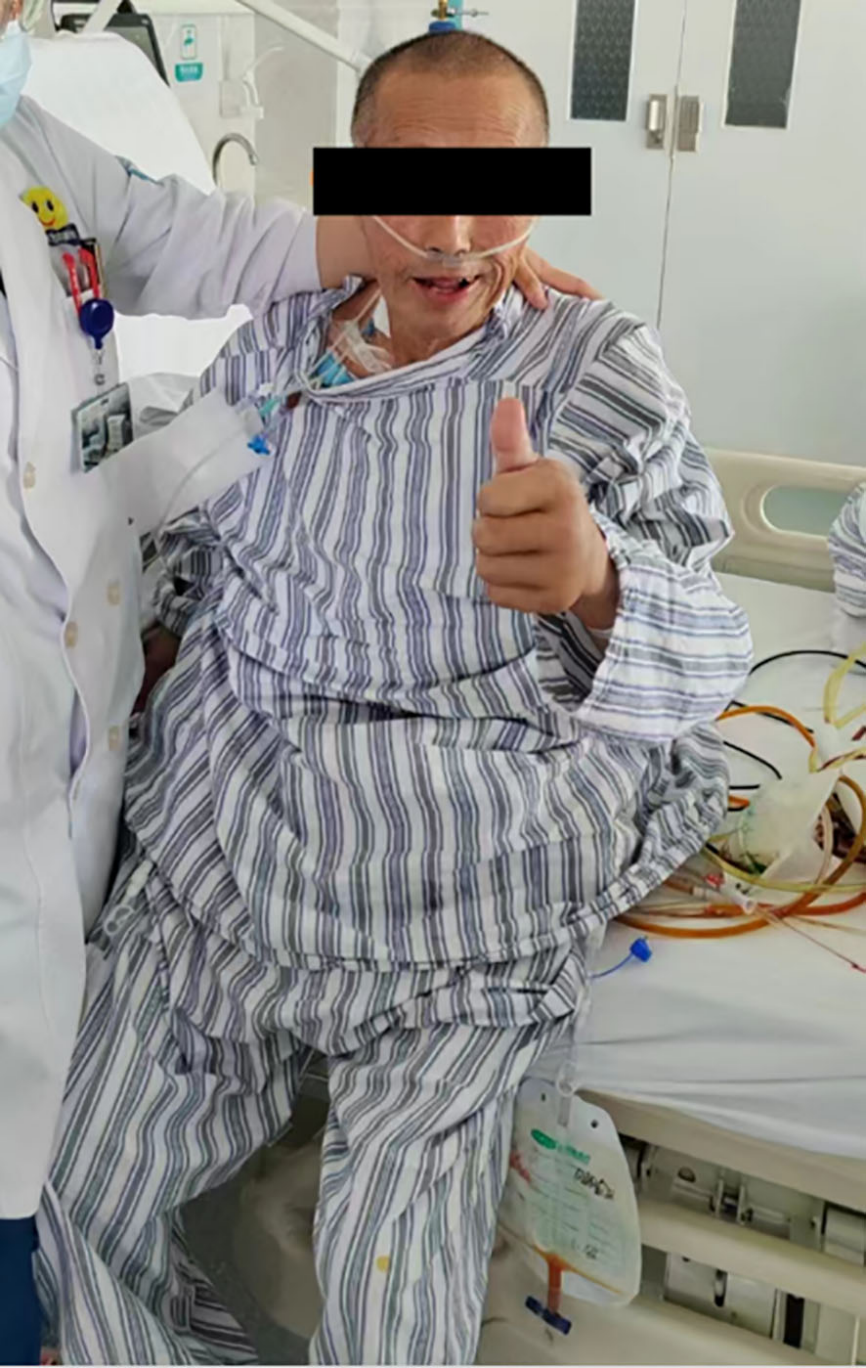
The patient was in good condition on POD 5.

Clinical Course of the Patient: The patient was weaned from ventilator support on postoperative day (POD) 3 and successfully extubated on POD 5, with stable vital signs throughout (**Figure 10**). A chest X-ray on POD 3 revealed bilateral lower lobe pneumonia with right pleural effusion, prompting immediate initiation of piperacillin-tazobactam for infection control and ultrasound-guided thoracentesis for drainage, supplemented with oxygen therapy and nebulization. However, the pulmonary infection exhibited a fluctuating clinical course, characterized by alternating periods of improvement and deterioration. On POD 15, the patient developed an ileocolonic anastomotic leak. Conservative management was initiated, including continuous gastrointestinal decompression, combined antibiotic therapy (piperacillin-tazobactam plus ornidazole), somatostatin infusion to reduce digestive secretions, maintenance of peritoneal drainage, and enhanced parenteral nutrition support. Unfortunately, after three days of conservative treatment, the patient’s condition deteriorated rapidly, manifesting septic shock (hypotension, tachycardia, high fever, and altered mental status). Laboratory findings revealed severe hypoalbuminemia (albumin 22.55 g/L), profound thrombocytopenia (4×10^9^/L), and anemia (hemoglobin 85 g/L). Following emergency blood transfusion and fluid resuscitation for shock management, an emergency ileocolonic fistula repair was performed. Intraoperatively, an everted, lip-shaped fistula was identified and repaired, with abdominal drainage tubes placed post-repair. The patient was subsequently transferred back to the ward in stable condition.

The patient’s outcome was as follows: Despite maintaining hemodynamic stability throughout the operative course and undergoing meticulous gastrointestinal reconstruction, the patient developed postoperative anastomotic leakage attributable to the confluence of extreme tumor dimensions, prolonged disease duration, significant surgical trauma, and subsequent intestinal wall edema. The ensuing 40-day clinical course was complicated by progressive pneumonia, decompensated heart failure, and abdominal sepsis, ultimately resulting in mortality. The Ethics Committee of Shanxi Provincial People’s Hospital has formally reviewed and approved this case report for academic publication and knowledge dissemination.

## 3 Discussion

RPLS is an extremely rare malignant tumor originating from retroperitoneal adipose tissue, accounting for 10%-15% of all soft tissue sarcomas, which in turn constitute only about 1% of adult malignancies (6). The unique anatomical features of the retroperitoneal space enable these tumors to grow to large sizes and often invade multiple adjacent organs. Compared with liposarcomas in the extremities, retroperitoneal ones exhibit more aggressive biological behavior and pose greater surgical challenges for complete resection (1).

Histologically, the well-differentiated subtype is predominant, characterized by tumor cells similar to mature adipocytes with mild atypia and rare mitotic figures. Despite their relatively indolent histological appearance, these tumors often infiltrate extensively along tissue planes, leading to tight adhesions with surrounding structures. At the molecular level, amplification of MDM2 or CDK4 genes is a hallmark feature. The MDM2 protein binds to and inhibits the function of the p53 tumor suppressor, thus promoting tumor progression (1). In this case, fluorescence *in situ* hybridization confirmed MDM2 gene amplification, providing definitive molecular evidence for the diagnosis.

Surgical resection remains the main treatment for RPLS. When technically possible, complete R0 resection should be pursued, as it has consistently been associated with better survival outcomes and lower recurrence rates compared to R1 resection. However, the frequent involvement of adjacent organs and vital structures often requires multivisceral resection to achieve clear margins. Current evidence regarding the benefits of such aggressive resections is conflicting. Some studies have shown that the 5-year survival rate of R0 resection is significantly better than that of R1 resection (p < 0.05), and the prognosis of patients has been better improved (7, 8). However, other studies have found that the probability of relevant complications occurring postoperatively in patients who received R0 resection is significantly increased. Despite the more aggressive surgical approach, there has been no significant improvement in terms of survival advantage (9, 10). This clinical dilemma highlights the importance of careful patient selection and individualized surgical planning to optimize the risk-benefit ratio for each case.

Therefore, when achieving an R0 resection is challenging, an R1 resection can be considered a viable alternative (11). Compared to an R0 resection, an R1 resection is less invasive and has a lower incidence of postoperative complications. A study by Paik et al. (12) involving 552 patients with retroperitoneal sarcoma (RLS) compared the complication rates between the R0 and R1 resection groups. The study showed that the surgical complication rate was 12% in the R0 group and only 2% in the R1 group, indicating that R1 resection is safer. For patients with incomplete resection of giant tumor masses, several retrospective studies on postoperative adjuvant radiotherapy have shown that it may reduce the recurrence rate. Additionally, combined incomplete resection and targeted therapy represents a potential treatment option, particularly for those with MDM2 overexpression and CDK4 overactivation, in whom targeted therapy is clinically feasible (13). Furthermore, anti-VEGFR targeted therapies such as anlotinib capsules hold promise in the later-line treatment of sarcoma (14).

In the present case, an R0 resection combined with multivisceral resection was ultimately performed. Although R0 resection is the optimal goal in treating RLS, the occurrence of anastomotic leakage in this case raises several relevant questions. These include whether an aggressive R0 resection should take precedence for large tumors and whether repeated surgeries increase the morbidity burden on patients. Moreover, in certain cases, prioritizing symptom relief over long-term survival may be worth further consideration. Given that multivisceral resection may bring additional risks, clinical decision-making should involve a thorough assessment of the balance between the benefits and risks of surgery, aiming to formulate an optimal and individualized treatment plan for each patient.

In recent years, the comprehensive treatment strategy combining cytoreductive surgery (CRS) with HIPEC has become a significant therapeutic modality for primary peritoneal malignancies (15). The main aim of CRS is to achieve the most extensive resection of macroscopically visible tumor tissues in the abdominal and pelvic cavities, including the involved organs (16). The surgical approach in this case strictly adhered to this principle, and HIPEC was administered after CRS to eliminate residual cancer cells and microscopic lesions in the peritoneal cavity.

However, the clinical significance of HIPEC in treating retroperitoneal sarcomas remains controversial. Seo et al. (17) reported the outcomes of radical resection combined with HIPEC in six patients with RPLS. The results showed that three cases had recurrence and died of the disease within 6 to 24 months. These results suggest that although HIPEC is technically feasible, its effectiveness in improving survival outcomes seems limited. A retrospective study by Francisco et al. (18) involving 23 patients with RPLS treated with CRS+HIPEC between 2016 and 2022 demonstrated a 5-year overall survival rate of 64%, with an acceptable level of perioperative safety (severe complication rate: 13%). However, the study could not determine the independent contribution of HIPEC to survival prolongation. Taken together, these findings indicate that the therapeutic role of HIPEC in managing RPLS needs further verification.

It is worth noting that anastomotic leakage occurred as a postoperative complication in this case. Dong et al. (19) have shown that HIPEC results in an uneven temperature distribution in the abdominal cavity. Specifically, deeper regions have higher temperatures, while areas near the body surface have lower temperatures. Additionally, slight positional changes of the perfusion catheters can cause localized thermal injury, posing significant obstacles to anastomotic healing. Yang et al. (20) conducted a meta-analysis of four studies and found that non-hyperthermic intraperitoneal chemotherapy significantly increased the risk of anastomotic leakage ([OR] = 2.05, 95% [CI]: 1.06-3.98). Although HIPEC was not analyzed separately in their study, these findings imply that hyperthermic perfusion may hinder anastomotic healing through local thermal stimulation. In this case, while the prolonged compression by the massive tumor might have caused tissue edema and a suboptimal preoperative nutritional status could have affected the healing process, the satisfactory surgical closure and normal preoperative protein levels suggest that the thermal effects and local stimulation of HIPEC likely had a negative impact on the anastomotic recovery after extensive tumor resection. This observation emphasizes the need for a more cautious assessment of the potential effects of HIPEC on tissue repair, especially in patients who have undergone extensive resections.

Common complications after radical resection of RPLS include hemorrhage, anastomotic leakage, surgical site infection, and intestinal obstruction. All these complications significantly affect perioperative safety and long-term prognosis (21). In this case, the anastomotic leakage was mainly due to compromised local blood supply, excessive mechanical tension, and inflammatory edema. This complication can potentially lead to secondary abdominal infection, sepsis, and other severe clinical consequences, and it is an important independent risk factor for increased postoperative mortality (22). This case highlights the critical importance of implementing systematic strategies to prevent perioperative complications in high-risk surgical patients.

In recent years, bioadhesives have demonstrated unique value in strengthening gastrointestinal anastomoses, attributed to their excellent tissue compatibility, biodegradable properties, and physical barrier functions. By creating a mechanical sealing interface, bioadhesives provide more reliable protection for postoperative recovery. At the same time, they reduce the risk of infection by limiting the migration of pathogenic microorganisms (23). Especially in complex surgical procedures like multivisceral resection, bioadhesives can significantly enhance the physical barrier function and improve the microenvironment for tissue healing. This advantage was verified by an animal study by Wenger et al. (24). In a porcine model, the group treated with bioadhesives had no anastomotic leakage during the 21-day observation period, while the control group had a 20% anastomotic leakage rate (p<0.05). Based on the existing evidence, we suggest incorporating bioadhesives into standardized protocols for reinforcing anastomoses in high-risk patients undergoing multivisceral resection of large retroperitoneal tumors. This approach not only conforms to the principles of damage control surgery but also provides patients with multiple protective barriers. The experience of this case highlights the importance of establishing a multidimensional prevention system, including the application of biomaterials, for high-risk surgeries in special anatomical locations, which has significant implications for improving clinical outcomes.

This study has several limitations. Firstly, as a single-center case report, the study results may be influenced by individual differences and selection bias. Secondly, the patient’s death within 40 postoperative days was caused by multiple risk factors, including prolonged tumor-related cachexia, massive tumor compression, and significant surgical trauma. This makes it difficult to quantify the contribution of any single factor. Thirdly, due to the uniqueness of the case, we were unable to evaluate the long-term prognostic impact of the interventions. Nevertheless, the short-term adverse outcomes of this case still have significant clinical warning value. They highlight the need to develop more comprehensive risk assessment systems and personalized treatment strategies for patients with advanced giant retroperitoneal tumors.

## 4 Conclusion

The surgical treatment of RPLS faces many challenges. Firstly, the tumor’s aggressive growth pattern and frequent dense adhesions to key retroperitoneal blood vessels and adjacent organs greatly increase the difficulty of radical resection. Secondly, for giant tumors as in this case, surgeons need to carefully balance tumor en bloc resection and organ function preservation. Although achieving R0 resection is a crucial independent prognostic factor, the need for multivisceral resection to achieve this goal in our case, combined with the patient’s cachectic state due to the massive tumor burden, significantly affected surgical tolerance and postoperative recovery. Therefore, meticulous perioperative risk assessment is essential. This requires establishing a multidisciplinary collaborative approach to optimize surgical decision-making, carefully evaluate potential postoperative complications, and actively implement comprehensive prevention strategies, including the application of biomaterials. Moreover, the decision to perform intraoperative HIPEC in such cases should be evidence-based and carefully evaluated according to the patient’s condition and expected prognosis.

## Data Availability

The original contributions presented in the study are included in the article/supplementary material, further inquiries can be directed to the corresponding author/s.
